# ‘If her body responds then you know she wants it’: a qualitative study on how young people in Ecuador and Uganda understand and practice consent

**DOI:** 10.1080/16549716.2025.2465123

**Published:** 2025-06-26

**Authors:** Sara De Meyer, Katie Lau, Elizabeth Kemigisha, Ana Cevalllos, Lucia Rost, Kristien Michielsen, Anna Kågesten, Miranda van Reeuwijk

**Affiliations:** a Independent Consultant, Carahue, Chile; b Independent Consultant (Previously SRHR Advisor, Plan International), Madrid, Spain; c Department of Health and Wellbeing, African Population and Health Research Center, Nairobi, Kenya; d Faculty of Interdisciplinary Studies, Mbarara University of Science and Technology, Mbarara, Uganda; e Research Directorate, University of Cuenca, Cuenca, Ecuador; f Head of Research, Plan International, Bristol, UK; g Institute for Family and Sexuality Studies, Department of Neurosciences, Catholic University of Leuven, Leuven, Belgium; h Department of Global Public Health, Karolinska Institutet, Stockholm, Sweden; i Rutgers International, Utrecht, The Netherlands

**Keywords:** Maria Emmelin, Sexual consent, youth, Ecuador, Uganda, youth co-researchers

## Abstract

**Background:**

Although progress has been made, adolescent sexual and reproductive health and rights (ASRHR) remain a global public health concern and continued investments are necessary. The way young people perceive, and experience sexual consent is at the centre of free and informed decision-making, a critical element of ASRHR. Nevertheless, the evidence on young people’s views of sexual consent remains limited, especially in low- and middle-income countries.

**Objectives:**

To explore how young people (aged 18 to 26) understand and practice sexual consent in low- and middle-income countries.

**Methods:**

Qualitative in-depth interviews and focus group discussions were conducted among 173 young people in Ecuador (Guayaquil) and Uganda (Kampala city), involving young people in data collection and analysis. The Hickman and Muehlenhard’s (1999) framework was used to categorize the ways young people express sexual consent: direct and indirect verbal and nonverbal signals.

**Results:**

We found that reflecting on sexual consent was relatively new for many participants and challenging to define. Few young people seemed to know how to apply consent in their daily lives, explaining that asking consent was often not possible in their relationships and sexual encounters. Influencing factors such as gender norms, the type of relationship and age were mentioned. Non-direct, non-verbal consent were the most commonly used strategies, meaning that sexual consent was mostly interpreted from body language or indirect questions – opening a window for sexual risks.

**Conclusion:**

Our findings call for SRHR programming that promotes communication and equality and supports skills in recognizing, giving and receiving sexual consent.

## Paper Context

- **Main Findings:** We found that reflecting on sexual consent was relatively new for many participants and challenging to define. Few young people seemed to know how to apply consent in their daily lives, and the respondents illustrated how asking consent was often influenced by traditional gender norms, the type of relationship and age.
- **Added Knowledge:** Our findings contribute to the knowledge on how young people living in low- and middle-income countries perceive sexual consent.
- **Global health impact for policy and action:** This study indicates an urgent need for SRHR programming to enhance the ability of young women and men of various ages to understand, recognise and respect sexual consent in order to both prevent or avoid sexual activities that significantly increases the risk of harm, and to engage in safe and positive experiences.

## Background

Since the International Conference on Population and Development (ICPD) in 1994, adolescent sexual and reproductive health and rights (ASRHR) has been gaining importance on the global agenda [1]. The funding for programmes and research-targeting adolescents has increased, and the number of evidence informed policies, normative documents, and guidelines on adolescent-responsive sexual reproductive health and rights (SRHR) programming have substantially grown [1,2]. These investments have contributed to more positive ASRHR outcomes. For example, early marriage has significantly declined in the 25 years after ICPD, and adolescent girls are more likely to use contraceptives [3]. Despite this progress, ASRHR remains a global public health concern and continued investments are necessary.

Half of all adolescents grow up in multi-burden countries characterized by high fecundity and an unmet need for contraception, particularly among unmarried, sexually active adolescents [4]. In Ecuador, 18.8% of all registered births in 2018 were among adolescents between the age of 15 and 19 [5]. In Uganda, at least 25% of women became pregnant before age 18 and adolescent pregnancy contributes to 17% of the overall maternal mortality [6]. Although 18 years is the legal age of consent for men and women to engage in sex in Uganda, a study in an informal settlement of Kampala found that the median age of first sex was 16 years and 31% of these experiences were non-consensual encounters [7,8]. The legal age of consent for sex in Ecuador is 14 [9].

A conceptual framework for healthy adolescent sexuality developed by Kågesten and van Reeuwijk (2021) describes safe and consensual sex as a critical part of positive sexual wellbeing for adolescents [10]. The way young people perceive, and experience sexual consent is important for their sexual relationships, wellbeing, health, and development as it is at the centre of free and informed decision-making, a critical element of SRHR [11]. A review by Fenner (2017) shows a lack of coherence and consistency in the representations of sexual consent among young people [12].

Past research in high-income countries (United States, Sweden and the UK) indicates adolescents find it easier to define sexual consent in abstract situations than in real-life situations. Such abstract definitions of consent were influenced by legal allowances and affirmative consent policies (yes versus no), reflecting consent as a mutual agreement at a specific point in time [13]. However, in real-life situations, young people faced contradictory norms, showing how meanings of sexual consent have gendered, historical, cultural and social roots [14]. For example, in many contexts, social and gender norms prescribe that young women should submit to men’s desires, to be pleasing, attractive, sexually available, and nurturing to men [12,15–17]. These gender norms can have implications on the possibilities, the desires and the agency of girls and young women to consent or to practice safe sex and negotiate for condom use [12,17–19].

Despite the challenges in defining sexual consent, young people consider it very important [13,17,20–22]. Fenner’s review of studies in English-speaking countries, including the US, Canada, New Zealand and Australia, found that young people perceive verbal consent as awkward and unnatural [12]. While they see the benefits of clear verbal consent, research shows that consent is often communicated nonverbally or by not resisting sexual advances [13,23–25]. Communication about sexual consent tends to be indirect (e.g. asking ‘Do you want to come over tonight?’), using a mix of verbal and nonverbal cues [12,13,26].

These findings highlight a research gap, as most previous studies focus on North America and Western Europe, and do not represent young people in all their diversity. Less is known about sexual consent among young people in low-and-middle-income countries [12,27].

To inform their ASRHR interventions on the basis of young people’s lived realities and needs across different continents, Plan International commissioned this study focusing on young people living in Ecuador and Uganda, two countries with significant needs related to ASRHR. We aimed to explore how young people in Ecuador and Uganda define sexual consent and to describe how they experience it in their daily lives.

## Methods

### Study design and setting

We conducted a qualitative study between August and September 2021 among young people living in Ecuador (Guayaquil, Nueva Prosperina and Socio Vivienda parishes) and Uganda (Kampala city, informal settlements of Katoogo and Kakungulu). The settings were selected because they are located in a low- (Uganda) and middle- income (Ecuador) country, and allow to explore how different socio-cultural and political contexts influence young people’s realities and experiences around sexual consent. The study was commissioned by Plan International, and implemented in collaboration with Rutgers, Karolinska Institutet and country research teams. The existing partnerships between Plan International and communities in the study settings thus permitted to integrate recommendations from the research into local SRHR programming.

### Study participants and data collection

For data collection, the country research teams were led by an international researcher and a national researcher, both experienced in qualitative research. In Ecuador, the team utilised the ‘Explore Methodology’ developed by Rutgers, integrating six young co-researchers (three young women and young men, 17–22 years) from the study areas [28]. The co-researchers were trained and involved in conceptualisation, data collection and analysis. They collaborated with Plan International Ecuador staff to recruit the participants and conducted the IDIs and FGDs. Working in pairs, one co-researcher led the interviews, while the other took notes, all in Spanish. The national researcher (from Cuenca) participated in some IDI and FGD as support for the co-researchers. All researchers and participants were native Spanish speakers. For clarification of regional Spanish terms, the national researcher requested support from the co-researchers.

Collaborating with young co-researchers helped to reduce power differentials, increase credibility and dependability, and to create a safe and open environment [29]. In data analysis, young co-researchers generated different discourses from adult researchers, using language and methods that better fit their peer’s knoweldge and interests [30]. Furthermore, their experiences and understandings of growing up in the research areas also informed the research design and data interpretation.

In Uganda, the IDI and FGD were conducted in English and Luganda. Research assistants who were fluent in Luganda assisted the researchers during data collection with translation. Due to strict COVID-19 mitigation measures, it was not possible to work with young co-researchers. The Ugandan team included three research assistants aged 25–28 years and was supported by an expert panel of six young experts (four young women and two young men, 18–26 years). Panel members were selected because of their previous work experience with young people on SRHR, providing insights on the data collection process as well as the interpretation of findings. Four online meetings were held with the Uganda young expert panel to receive feedback, which enhanced the quality of the research and its outcomes.

We conducted a total of 21 FGD (10 in Ecuador and 11 in Uganda) and 54 IDIs (27 in each country) which allowed us to reach data saturation. In both countries, the primary research participants were young people aged between 18 and 26 years old, who responded retrospectively to questions about their experiences from the age of 10 years. In Ecuador, 12 young women and 15 young men participated in the IDI, and 28 women and 25 men joined in the FGD. In Uganda, 15 young women and 12 young men were interviewed and 34 young women and 32 young men participated in the FGD. The co-researchers and young experts were also considered participants and interviewed in English by the international researcher. Purposive sampling was used in both countries to select participants based on the following criteria: age (18–26), equal distribution between sex assigned at birth, and residence in the selected areas. In Ecuador, the participants were personally contacted by the co-researchers, who lived in the selected communities, with support from Plan International Ecuador. In Uganda, the respondents were invited to participate by community mobilizers from Plan International Uganda. The IDI focused on personal experiences related to sexual wellbeing and sexual consent, while the FGD focused on more general experiences of young people in relation to these concepts. The former lasted on average one hour while the latter took on average one hour and a half. The guides were developed in English and translated into Spanish (Ecuador) and Luganda (Uganda).

### Analysis and conceptual framework

All interviews were audio recorded and transcribed by native speakers in Spanish and English from Ecuador and Uganda, respectively. Data were coded and organised into themes and categories, based on the framework developed by Kågesten and van Reeuwijk on adolescent sexual wellbeing, using NVivo 12 [10].

For the analysis of how young people express sexual consent, we used Hickman and Muehlenhard’s four types of consent as the conceptual framework: (1) direct verbal signals, (2) direct nonverbal signals, (3) indirect verbal signals, and (4) indirect nonverbal signals [14]. We applied this framework to the study findings to explore how young people in Uganda and Ecuador practice consent in their daily lives. During the analysis, we paid special attention to unpacking differences based on gender and age. All transcripts were coded by the national lead researchers and about half of the transcripts were also coded by the international researcher. The codes were compared until a final set could be defined. A deductive approach was applied for coding.

### Ethics

The study was approved by the ethical committees from the University of Cuenca (Ecuador) (2021-0111EO-I) and Mbarara University of Science and Technology (Uganda) (MUST-2021-37). In Uganda, additional approval was obtained from the Uganda National Council of Science and Technology (HS1438ES). In both countries, administrative clearance was also provided from the community leaders in the areas where data collection took place. Before participating in the research, all respondents were asked to give written informed consent. Ethical approval was also granted by the Swedish Ethical Review Authority (EPN) (2021-06,564-01) for analysis of de-identified data.

All researchers and data collectors participated in Plan International’s Safeguarding training and worked closely with Plan International Safeguarding focal points in the countries to ensure that risks were mitigated, and safeguarding concerns were adequately addressed. We chose the respondent age range to consider national laws around the age of sexual consent and to increase the likelihood that young people would feel both comfortable and able to discuss personal experiences related to sexual consent.

## Results

### Defining sexual consent

#### General definition

Sexual consent appeared to be a new concept for most of the female and male participants. The majority of the respondents in both countries found it difficult to define key aspects of sexual consent and how it is obtained.

I have just heard of sexual consent today. It is my first time. (Uganda, FGD 10, young women only, 18–22 years)

Young people appeared to have their own understanding of consent – not a formal yes or no – with focus on indirect actions. When sexual consent was defined, it seemed to be interpreted on a spectrum. The most common interpretation of consent was active agreement to have sex, or to respect each other’s boundaries, in one way or another.

It depends on both parties: when they agree as couples to have sex, that is what we call a good relationship, and it means that there is consent. (Uganda, FGD 2, young men only, 18–22 years)

[Sexual consent means] your partner agrees to have sex with you. (Ecuador, FGD 1, young men only, 18–20 years)

Respect my choices. For example, there are people who like anal sex and others who don’t, but you have to respect. If I don’t like it, you respect it. (Ecuador, young woman, 19 years)

On the other hand, some young people indicated that consent was the result of compromising, and that, at times, the mere absence of refusal equalled consent.

If the boy touches the girl and she does not resist, it means she has accepted and she has allowed the boy to do whatever he wants. (Uganda, FGD 1, young women only, 18 years)

#### Different signals to express sexual consent

Besides a general description of sexual consent, young people gave examples of situations in which various signals could be used to express sexual consent. We categorise these examples, using the four strategies that were identified by Hickman and Muehlenhard to communicate sexual consent [14].

##### Direct verbal signals

In both countries, most of the respondents felt that asking for consent directly and verbally was difficult and made them feel embarrassed. We found that direct verbal signals to communicate sexual consent were rarely used and when they were, it was mainly by boys and young men.

Yes, there is a difference, because the man comes up to you and says: “You know what, I want to be with you”, and men are like that, they just say it; but the woman won’t come up to you and say “I want to be with you” because she is embarrassed. It’s more the man who invites. (Ecuador, FGD 2, young women only, 18–20 years)

According to the respondents, young men’s use of direct approaches was closely related to their age, noting, for example, that older men were more likely to ask (young women) for sex more directly than younger boys do.

I would think that age would come into play here. If it’s an older person, let’s say he’s going to ask the girl if she wants to have sex, but if it’s a teenager, they focus more on watching Netflix or things like that […] In this case, if the girl does not want [to have sex], she will just say “I’ll go with you” but in the end she stands him up and doesn’t go, then that would be her way of saying she doesn’t want to [have sex]. (Ecuador, meeting of co-researchers, 17–22 years)

However, young participants in Uganda mentioned that young women can say ‘no’ but that this may not always be accepted.

R1: For a girl’s no to be a no, it has to be accompanied by reasons. In most cases, girls don’t say yes, they say no.

R2: Also the tone matters.

R1: Even the tone may be high but still the no meaning yes except if she accompanies her no with reasons. (Uganda, FGD 3, young men only, age 21–23 years)

Moreover, young women are expected to say ‘no’ or resist in another way, whether they like the young man or not. The young women’s hesitancy to say ‘yes’ immediately if interested in sex may be attributed to maintaining value or dignity.

##### Direct non-verbal signals

Direct non-verbal signals were the most acknowledged signals among the respondents in both countries. According to the participants, these were gestures or physical actions that could be interpreted as encouraging for the partner to continue the sexual behaviour. These included a response (mainly from young women to a touch/caress, or a response to a kiss and so on (from a young men).

I mean, it is very likely that men, like women, become flirtatious, giggly, or with their looks, or in the way they talk… . (Ecuador, FGD4, young women only, 19–21 years)

I mean it’s the flirting, the touching, they might touch your back, before they used to hug you but now they’re touching your buttocks or they might start to get a little more romantic, they start to give you more affection, it’s more sticky, it’s more chewy. (Ecuador, FGD 3, mixed group, sex unknown, 18–21 years)

Respondents described that if a partner does not respond to the touch or caress, or brushes it off, this is a form of non-verbal signal that they do not give consent to sex. Other direct non-verbal signals for non-consent described include body language such as turning their back to someone or sitting cross-legged.

##### Indirect verbal signals

Using indirect verbal signals means that young people ask a question which implicitly asks for a sexual encounter. Respondents described various indirect verbal signals in the form of informal cues which in turn could easily be misinterpreted. In Uganda, young people mentioned that they could ask for consent by inviting someone to come and visit them at home. Girls and young women would be considered to be indirectly consenting when visiting a boy or young man at home or by ‘*saying no in a soft tone or whisper*’ or asking to take a bath at his home.

But a girl who is going to give you some [sex]… She can ask to even bathe from your place, all those are signs. (Uganda, FGD9, young men only, 21–24 years)

In Ecuador, young people might invite each other to watch Netflix. If someone accepted these invites, sexual consent was implicitly assumed.

They invite you to see a movie … [But] … you already know what is going to happen. You know it is [being] home alone, so the movie never ends up being a movie [but also having sex]. But they [the young people] know the meaning [of being asked to watch a movie]. (Ecuador, meeting of co-researchers, 17–22 years)

In addition, our findings pointed to the indirect question for ‘la prueba de amor’ [proof of love], commonly asked by young men in Ecuador to their girlfriend and which refers to engaging in sexual behaviour. Female participants noted that it can be difficult for young women to refuse (unwanted) sex because they were afraid of upsetting or losing their partner.

Boys ask for the “proof of love”. If you don’t give him the “proof of love” [have sex] it’s because you don’t love him. So, I have seen among my friends that if they don’t give him [the boyfriend] the proof of love, they [the boyfriends] leave them, and the girl gives him the “proof of love” for fear of being left alone. (Ecuador, young woman, 21 years old)

Sometimes the woman is like, “well, if I tell him that [no to sex] he is going to leave me for someone else”, she creates insecurities in her mind and sometimes she accepts [having sex] because of that. (Ecuador, young woman, 18 years)

Young respondents in Uganda also illustrated how indirect verbal signals can be applied to convey non-consent.

I: So what other ways can she show that she is not interested.

R1: They can claim to be in their periods.

R2: Others can say they are sick. (Uganda, FGD9, young men only, 21–24 years)

##### Indirect non-verbal signals

Participants in both countries referred as well to indirect, non-verbal ways of asking for consent, which was primarily what young women do to implicitly reveal interest or lack of interest in having sex. There were no examples described in terms of how young men would indicate consent or non-consent. In Uganda, giving and accepting gifts was considered the most important approach, meaning that if a young woman accepts a gift from a young man, sexual consent is perceived to be given. Other examples discussed included young women behaving sleepy or shy and young men appearing sweaty and restless due to being sexually stimulated. If a young woman dressed ‘provocatively’, befriended the young men’s siblings or parents, or comes to his home, this would also be interpreted by some as consent for sex.

The phone calls, surprises and gifts which are exchanged show sexual consent. When a girl takes your gifts, it means she has already consented to be with you, even sexually. (Uganda, FGD 6, young men only, 22–24 years)

Young Ugandan participants also described that to indirectly say ‘no’ in a non-verbal way, girls and young women could bring friends when visiting a boy or young man, keep their visits very short, or simply avoid picking up the phone or visiting at all. In all these cases, the respondents noted that the boy or young man will know that sexual activity is out of the question.

Participants in Ecuador illustrated how text messages such as ‘are we going to make a baby’ or ‘are we going to kill it [the sexual tension]’ or messages with aubergines and apricots were sent to invite someone to have sex. It seemed that, in contrast to real-life experiences, young women were socially less restricted from taking the initiative online.

Nowadays, … everything is done in the chat room, and they agree on something. For example, a girl said to my cousin: “yes, tomorrow I’ll wait for you to have a baby”, but not to get her pregnant, just to be intimate. (Ecuador, FGD 2, young women only, 18–20 years)

I think I would tell him by message, but after a month I wouldn’t be able to see him. He would say: “she is shy, reserved”. It’s that by message you are one but personally you are another, because by message I only write, and they don’t see my face. (Ecuador, young woman, 19 years old)

## Practising sexual consent

As mentioned before, the term sexual consent seemed to be new for most of our participants. In addition, few of the young respondents seemed to know how to apply it explicitly in their daily lives, explaining that obtaining sexual consent was often not possible.

According to people of our age, it is not easy to seek consent; some boys will just start the action without asking you for consent. So, I think some young people do not understand it properly. (Uganda, FGD 4, mixed group, sex unknown, 19–23 years)

It is also not about culture: a girl says no so that you can stick to her and take time chasing after her. (Uganda, FGD 3, young men only, 21–23 years)

Apparently, most young people did not consider the legal context when deciding to engage in consensual sex. Participants in both countries mentioned that communication about sexual consent does not happen that much in their community. Although this was not put in practice by all, many participants from both countries explained that boys and young men are usually expected to take the initiative for sexual activity, whereas girls and young women are the ones who need to decide where it stops.

No, it’s usually the boys who take the initiative. (Ecuador, meeting of coresearchers, 17–22 years)

They used a phrase that says, “hands to the prey”, that is to say that they don’t ask her directly, but they immediately start touching the girl to see what her reaction is, to see if she wants to or not. (Ecuador, meeting of co-researchers, 17–22 years)

Furthermore, young people in our study described that verbally indicating boundaries is very rare and that sexual consent was mostly assumed.

R1: In the sexual sphere, to have sex, I haven’t said anything, it just flows, and the sex happens. At least what I have experienced.

I: Maybe with expressions or words?

R: No, it’s like typical, we’re talking in the room, the kissing starts, and the temperature starts to rise, and it happens. (Ecuador, young man, 21 years)

R2: A girl may come and visit you, and if her body is down, you will know but if her body responds then you know that she wants it. …

R4: Most women have no programme, by the time you remove the knickers, she has already accepted. (Uganda, FGD 9, young men only, 21–24 years)

In addition, participants in both countries indicated that young women can genuinely also be interested in having sex and sexual pleasure.

Because when I want and you are not there to do what I want, I will go for another man. (Uganda, FGD 4, mixed group, sex unknown, 19–23 years)

Nevertheless, young people mentioned that sexual abuse and violence occurred quite often. In Uganda, participants indicated that young women can be harassed verbally or even physically.

He always wanted [sex]. … He would hurt me since it was forceful [sex]: my vagina would hurt, my legs would hurt, my chest would hurt and my whole body would hurt me. (Uganda, young woman, 22 years)

To some extent, violence also seemed justified by young people in Uganda based on participants narratives. There was a general perception that young men are simply entitled to sex if they provide gifts or money to a young woman in a relationship, or if a young woman visits their home. In such circumstances, the young women may be blamed if a forced encounter occurred during the visit.

If a girl visited and got raped, they would ask what she had gone to do there. Visiting a boy loosely translates to consent and so people would not pity the girl for getting raped. But it is not a must that every time you visit a man, there must be sexual relations. There are times when I just want to spend time with you being playful without anything sexual happening. (Uganda, FGD 11, mixed group, sex unknown, 18–23 years)

Also in Ecuador, participants indicated that these types of abuse occurred. Verbally, girls and young women, for example, receive sex-related comments from boys and men while growing up and experiencing body changes. One participant shared her story about physical abuse from her ex-boyfriend.

R: The one I was with, I mean, I didn’t give it [sex] to him. We were chatting and he was telling me that he wanted to be with me, I was thinking about being with him. And it was the other way, and we went out and then it happened, I didn’t want to.

I: When it happened did you express to him in any way that you didn’t want to at that time?

R: Yes.

I: How did you express yourself?

E: I told him I didn’t want to, out of fear. […] I was clear with him that I didn’t want to. But nevertheless, it happened. (Ecuador, young woman, 20 years)

However, the ability to express sexual consent was not only influenced by existing gender norms. It also seemed to differ between different types of relationships. Serious relationships could also lead to more respect and ability to (not) consent. There seems to be a direct relationship between ‘love’, consent and sexual wellbeing, whereas in ‘casual’ sexual encounters the goals seem to be different and more short-term and self-centred, for example, around pleasure, economic incentive and/or self-esteem, with less interest in the wellbeing and consent of the other.

In casual relationships, aspects of care are not taken into account. These measures are not used. But because of emotion [desire] they just go and do it [have sex], and they do it without protection. (Ecuador, young man, 18 years)

In addition, some respondents referred to the influence of age and mentioned that indicating boundaries becomes easier when older and more mature, with older being more informed, experienced and less naïve.

Fourteen year olds are just going through puberty, and what you hear is that there are many children who at an early age already want to have sex, so it’s like they are just kissing and kissing and end up doing what they shouldn’t, but at an older age you become aware and you have more experience. (Ecuador, young woman, 18 years)

## Discussion

Unlike existing research conducted primarily in Western Europe and North America, our findings revealed that reflecting on sexual consent was relatively new for young people in Ecuador and Uganda. The respondents found it difficult to define what sexual consent is. Among those who did, most provided a definition that was more abstract than applicable to real-life situations, consistent with the literature [13,21,31].

As such, our study confirms former research which indicates that sexual consent among young people is far more complicated than ‘yes’ or ‘no’, and is rarely a direct verbal signal [13,21,22,31]. When discussing real-life situations, sexual consent was often expressed differently in in terms of giving and receiving. Our findings show that the most frequently mentioned signals were direct non-verbal cues in both countries. This suggests that young people rely on social cues to indicate consent rather than explicit verbal communication, leading to assumptions and potential misinterpretation. Respondents often cited examples of sexual consent where it was unclear if consent was explicitly asked for or given.

Participants also confirmed the difficulty in refusing sexual activities. In some situations, young women lacked autonomy in decision-making, becoming victims of violence and rape. The perceptions that they could not always decline sex is closely related to how sexual activities are initiated, and to who is socially allowed or expected to do so. This highlights that understanding and practicing sexual consent is influenced by diverse contextual factors – affirming its complexity.

Our research identified various factors influencing the process of sexual consent. Consistent with Muehlenhard et al. and Oware et al., we observed that young people navigate heterosexual social scripts dictating sexual relations for young women and young men [13,31]. As noted in numerous studies – including in Ecuador and Uganda – girls and young women are often socially expected to assume passive roles, while boys and young men are expected to be sexual initiators and decision-makers [27,32–34]. The expectation pressures young men and simultaneously diminishes the agency of girls and young women. These restrictive scripts lead to misunderstandings and limit the autonomy to freely consent to sex, as shown in a 2023 study on sexual consent communication among young people in Nairobi’s informal settlements [31].

Our findings show that boys and young men are more likely to express sexual consent freely and to use direct verbal signals compared to girls and young women. At the same time, respondents did not mention situations where boys and young men did not consent, reinforcing the idea that they always want sex and are allowed to be sexually active. Conversely, girls and young women often feel unable to express their desires. Similar to the findings from Nairobi, traditional scripts of sexual chastity require young women to initially deny sex to avoid showing sexual interest [31], leading to genuine refusals being misinterpreted as consent. Young women’s actual refusals had to be assertive and accompanied with explanation.

However, our results also indicated an economic aspect of sexual consent. Similar to the findings among school pupils in southwestern Uganda, there is a perception that boys and young men are entitled to sex if they provide gifts or money [34]. Young women often initially deny sex to increase their bargaining value. These examples illustrate how gendered expectations influence young women’s behavior, reducing their voice and complicating the process of sexual consent.

Nevertheless, it is also important to point to changes in this narrative. Some young men in our study resisted the role of the sexual initiator, and some young women expressed their desires, particularly in online and digital spaces. The relative anonymity and perceived safety of the digital space may allow girls and young women to act without the inhibitions imposed by social norms.

Beyond gender norms, our findings highlight other factors affecting an individual’s ability to consent. Similar to the existing literature among 13 to 15 year-olds in the UK and Sweden, our research indicates that older age is positively associated with the ability to give sexual consent [17,21]. This suggests that experience, knowledge, and evolving capacities are important competencies, with older age correlating with increased ability to consent. Additionally, there seems to be a direct relationship between ‘love’ and consent, whereas in ‘casual’ sexual encounters, goals are more short-term and self-centred, focusing on pleasure, economic incentive and/or self-esteem, with less interest in the other’s consent.

Our findings highlight the urgent need for SRHR programming, such as comprehensive sexuality education that focuses on understanding positive, consensual, pleasurable and safe sexual experiences, and the importance of expressing, recognizing and respecting boundaries. Comprehensive sexuality education has been shown to improve sexual and reproductive health outcomes and gender equitable attitudes, but evaluations rarely focus on understanding and respecting for sexual boundaries and consent [35]. Instead, they often emphasize young women’ ability to ‘say no’, while our research findings indicate that rarely consent as simple.

Throughout the research, it was clear that lines of consent were blurred, and young people also mentioned frequent occurrences of sexual abuse and violence. Young people need the knowledge, confidence, and attitudes to make informed decisions, including consenting to sex in real life and in digital spaces. Plan International’s Standards for Content, Delivery, and Environment of CSE emphasize the need for a deeper understanding of sexual consent by both young men and women [36]. Our findings suggest the importance of working with realistic ‘scripts’ rather than (only) addressing consent in abstract ways, and to discuss power dynamics and gender inequalities as part of this. The data can help develop ‘scenarios’ to identify potential issues and ensure positive experiences. Such exercises can help promote alternative masculinities that embrace respect and caring, and introduce negotiation about contraceptive use into sexual scripts – providing young people with the language to do so and normalizing it as part of sexual communication.

A clear limitation of our research is that – due to the legal age of sexual consent – we focused on young people over the age of 18. This may have led to recall bias, as we asked about experiences of up to ten years ago. We took steps to mitigate its impact by inviting the participants to think about differences between ages. Furthermore, the vast majority (about 80%) of participants were recruited from Plan International’s programmes, and they may have been more assertive in discussing their experiences. We recognize that the selection of these participants could have introduced potential biases, including knowledge, self-selection, ethnocentric and social desirability biases. Additionally, while the involvement of young co-researchers may have helped participants to share information and improved the data analysis, it may have also limited the depth of information gathered. Furthermore, the immediate translation of Luganda audio into English transcripts may have led to some loss of information and nuance. Additional research involving a broader range of young people is needed to better understand how sexual consent occurs in the lives of young people in low- and middle-income countries.

## Conclusions

This study underscores the urgent need for SRHR programming to empower young people of all ages to understand, recognise and respect sexual consent. Such programmes should help prevent harmful sexual activities as well as promote safe, positive and pleasureable experiences. They should engage with young people in creating realistic, contextualised scripts to practice expressing and identifying wishes and boundaries, while critically addressing power dynamics and gender inequity. Evaluation of such programmes should include outcomes related to young people’s understanding, confidence and respect for sexual boundaries and consent.
